# OUTpatient intravenous LASix Trial in reducing hospitalization for acute decompensated heart failure (OUTLAST)

**DOI:** 10.1371/journal.pone.0253014

**Published:** 2021-06-25

**Authors:** Carine E. Hamo, Sahar S. Abdelmoneim, Seol Young Han, Elizabeth Chandy, Cornelia Muntean, Saadat A. Khan, Prasanthi Sunkesula, Marcella Meykler, Vidhya Ramachandran, Emelie Rosenberg, Igor Klem, Terrence J. Sacchi, John F. Heitner

**Affiliations:** 1 Division of Cardiology, Johns Hopkins University, Baltimore, Maryland, United States of America; 2 Division of Cardiology, Brooklyn New York-Presbyterian Hospital, Brooklyn, New York, United States of America; 3 The Duke Clinical Research Institute, Durham, North Carolina, United States of America; Prince Sattam Bin Abdulaziz University, College of Applied Medical Sciences, SAUDI ARABIA

## Abstract

**Background:**

Hospitalization for acute decompensated heart failure (ADHF) remains a major source of morbidity and mortality. The current study aimed to investigate the feasibility, safety, and efficacy of outpatient furosemide intravenous (IV) infusion following hospitalization for ADHF.

**Methods:**

In a single center, prospective, randomized, double-blind study, 100 patients were randomized to receive standard of care (Group 1), IV placebo infusion (Group 2), or IV furosemide infusion (Group 3) over 3h, biweekly for a one-month period following ADHF hospitalization. Patients in Groups 2/3 also received a comprehensive HF-care protocol including bi-weekly clinic visits for dose-adjusted IV-diuretics, medication adjustment and education. Echocardiography, quality of life and depression questionnaires were performed at baseline and 30-day follow-up. The primary outcome was 30-day re-hospitalization for ADHF.

**Results:**

Overall, a total of 94 patients were included in the study (mean age 64 years, 56% males, 69% African American). There were a total of 14 (15%) hospitalizations for ADHF at 30 days, 6 (17.1%) in Group 1, 7 (22.6%) in Group 2, and 1 (3.7%) in Group 3 (overall p = 0.11; p = 0.037 comparing Groups 2 and 3). Patients receiving IV furosemide infusion experienced significantly greater urine output and weight loss compared to those receiving placebo without any significant increase creatinine and no significant between group differences in echocardiography parameters, KCCQ or depression scores.

**Conclusion:**

The use of a standardized protocol of outpatient IV furosemide infusion for a one-month period following hospitalization for ADHF was found to be safe and efficacious in reducing 30-day re-hospitalization.

## Introduction

There continues to be a growing burden of heart failure (HF) due to rising prevalence coupled with extended, frequent hospitalizations with approximately one quarter of HF patients re-hospitalized within 30 days [1, 2]. HF management programs can reduce HF admissions by up to 80% with a multidisciplinary approach that provides early identification and intervention on symptom progression, patient education, and management of medical, socioeconomic, and psychologic factors that contribute to HF exacerbations [3–8].

Over the past several decades, several trials have failed to demonstrate effective treatments for acute decompensated heart failure (ADHF) that improve long-term outcomes [9]. Diuretics remain the mainstay therapy for HF symptom management with escalation of dosing as needed for decongestion. Several studies have shown that outpatient intravenous (IV) diuretic therapy is safe, cost effective, and may stabilize evolving deterioration and prevent hospital readmissions [1, 10, 11]. However, there have been no randomized controlled trials to date that utilize outpatient IV furosemide diuretic maintenance treatment in patients with HF and reduced ejection fraction (HFrEF) and HF with preserved ejection fraction (HFpEF) following hospitalization for ADHF.

The current study was a randomized controlled, double blinded study aimed to evaluate the feasibility, efficacy and safety of outpatient IV diuretic therapy in reducing 30 days re-hospitalization for ADHF following hospital admission.

## Methods

### Study design

OUTpatient Intravenous LASix Trial **(**OUTLAST) was a single center prospective randomized double-blind controlled trial. The study was approved by the Institutional Review Board of New York Presbyterian Brooklyn Methodist Hospital (Reference # 307139, approved 3/15/2012) and registered at www.clinicaltrials.gov (NCT04691687). The trial was registered after patient recruitment began due to an oversight as it was thought to have been registered prior to study initiation. The authors confirm that all ongoing and related trials for this drug/intervention are registered. All patients provided written informed consent.

### Study population

Adult men and women >18 years of age with a known history of systolic or diastolic dysfunction >6 weeks, New York Heart Association (NYHA) Class II-IV, elevated n-terminal-pro brain natriuretic peptide (NT- proBNP) ≥360 pg/ml not explained by any other etiology presenting to the hospital emergency department between May 2012 and June 2017 with at least one symptom (paroxysmal nocturnal dyspnea, orthopnea, dyspnea on mild or moderate exertion) at the time of screening and at least one sign (rales post cough, jugular venous pressure ≥10 cm H_2_0, lower extremity edema, or chest x-ray demonstrating pleural effusion, pulmonary congestion, or cardiomegaly) were screened and evaluated for study eligibility. Patients with a systolic blood pressure (SBP) <85 mmHg, signs of significant respiratory distress, biventricular intracardiac defibrillator (ICD) placement within 15 days, cardiogenic shock, acute renal failure or on chronic dialysis, severe systemic illness with life expectancy judged to be less than three years, chronic pulmonary disease requiring home oxygen, hemodynamically significant uncorrected valvular heart disease, or any valvular disease expected to lead to surgery during the trial, myocardial infarction in past 90 days, percutaneous coronary intervention in past 30 days, heart transplant or currently implanted left ventricular assist device, history of stroke in the past 90 days, allergy to lasix, known chronic hepatic disease, dementia or psychiatric illness, those blind or deaf and patients who were transferred to a different hospital were excluded.

### Randomization and intervention

**Fig 1:**
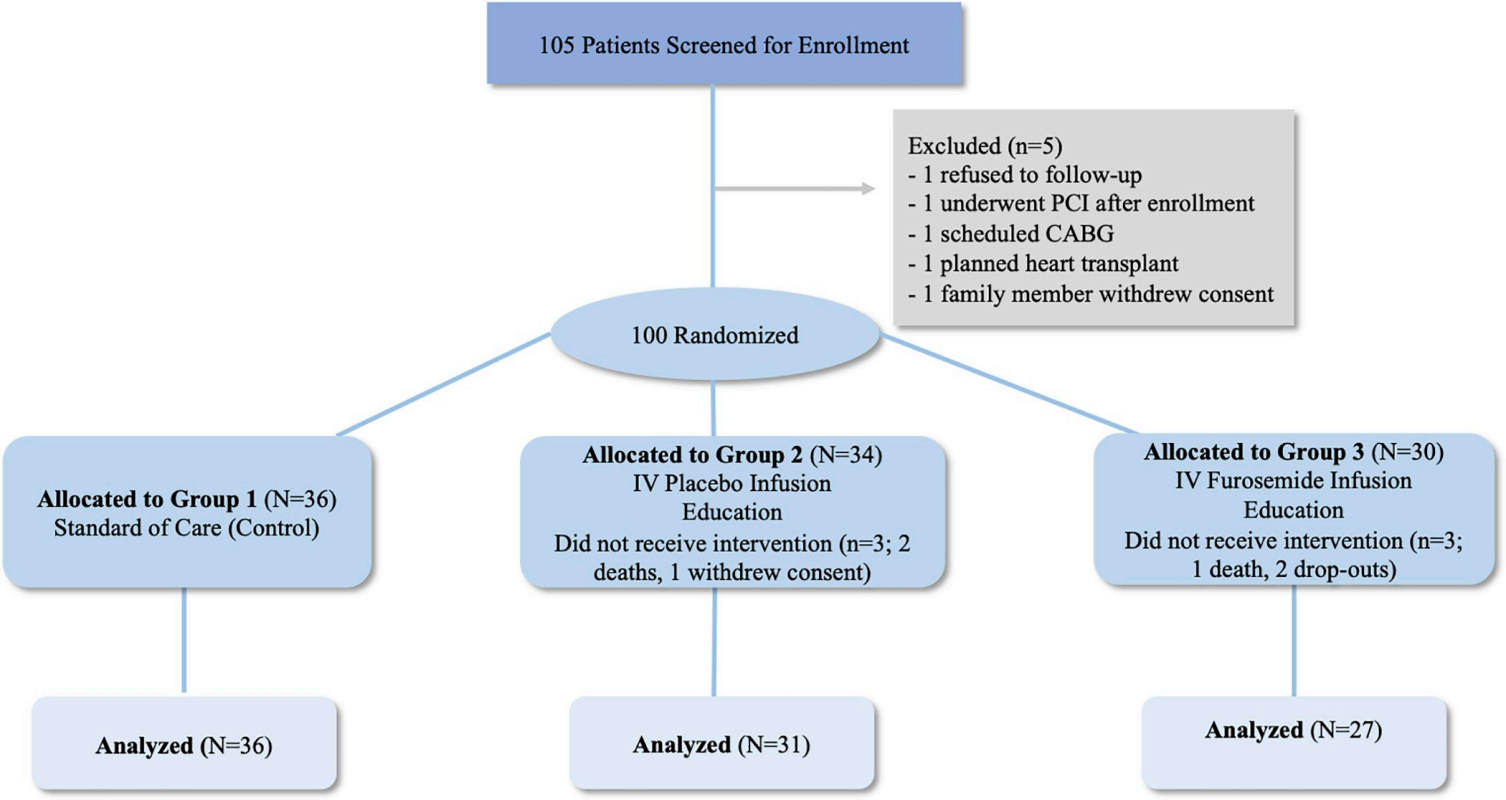
Overall study flow diagram. CABG, coronary artery bypass graft; IV, intravenous; PCI, percutaneous coronary intervention.

Patients were randomized by a clinical pharmacist with the ratio of 1:1:1 into 3 groups: standard of care control arm (Group 1), IV placebo infusion (Group 2), and IV furosemide infusion (Group 3). (**Fig 1**) Patients in Group 2 and 3 received a comprehensive HF-care protocol that included bi-weekly clinic visits for dose-adjusted IV-diuretics, medication adjustment and education. Patients, nurses and treating physicians were blinded to the randomization. Patients in Group 1 received standard of care treatment per heart failure guidelines at the discretion of the primary cardiologist involved in the patient’s care [8]. Patients in Group 2 received IV saline infusion (20–40 ml) concentrated by the pharmacist to minimize fluid intake. Patients in Group 3 received IV furosemide (LASIX, Sanofi-Aventis U.S. LLC Bridgewater, New Jersey) calculated by the pharmacist to be equivalent or higher in dose compared to the patient’s home oral dose. The dose assignments were categorized into low dose (20 mg bolus with 20 mg/hour infusion sessions and 2 ml saline), intermediate dose (40 mg bolus with 40 mg/hour infusion sessions and 4 ml saline) and high dose (80 mg bolus with 80 mg/hour infusion sessions). The infusions were continuous over 3h, biweekly over a one-month period. Infusions were held at the discretion of the physician utilizing a written protocol (creatinine 25% above baseline, SBP <80 mmHg or symptoms of presyncope). Patients in both Groups 2 and 3 resumed all of their oral home medications for HF post infusion visits per standard of care.

### Study visit monitoring

Patients randomized into either Group 2 (placebo/saline infusion) or Group 3 (furosemide infusion) were followed up at the HF outpatient infusion unit twice a week (8 visits per group per month; 464 total visits in both groups). Hemodynamic monitoring was performed during each study visit including weights at the start (prior to infusion) and end of the clinic visit. Fluid input and urine output were quantified during the study visits. Baseline laboratory testing (including basic metabolic panel and NT- proBNP) was performed for all study groups at baseline and 30-day follow-up, regardless of the treatment arm. Laboratory testing was done at the beginning and end of each infusion visit for Groups 2 and 3. Patients in all groups were monitored for any potential symptoms or side effects.

The infusion unit consisted of a multidisciplinary team that included a physician, pharmacist, and nurse. The infusion unit contained infusion chairs with cardiac telemetry, local medication storage, and infusion equipment. At each clinic visit, a detailed medical history was obtained, HF education material was provided, and medications were administered. A clinical pharmacist performed detailed medication reconciliation and evaluated medication adherence.

### Echocardiography

Echocardiography was performed at the baseline visit and one month following the baseline visit. Left ventricular ejection fraction (LVEF) was calculated using the modified Simpson’s method. The left atrial (LA) volume was calculated using the biplane area-length technique. The LV end-diastolic diameter (LVDd), LV end-systolic diameter (LVDs), septal wall thickness (SWT), and posterior wall thickness (PWT) were measured using M-mode echocardiography [12]. Diastolic function was assessed using transmitral and tissue Doppler imaging at the septal mitral annulus. Peak early (E) and late transmitral filling velocities (A), E/A, the deceleration time of peak E velocity, early diastolic mitral annular velocity (e’), and E/e’ were measured. Right ventricular systolic pressure was quantified from the peak tricuspid regurgitation velocity based on the Bernoulli equation and factoring in right atrial pressure which was estimated from the inferior vena cava (IVC) diameter and the collapsibility index [12]. Patients were classified as HFpEF if LVEF was >45% or HFrEF if LVEF was ≤45% on baseline echocardiography.

### Quality of life and depression assessment

Quality of life and depression were assessed at baseline and at 30 days using the Kansas City Cardiomyopathy Questionnaire (KCCQ) and the Depression Scale Health Questionnaire (PHQ 9). The KCCQ is a validated, 23-item self-administered questionnaire for the evaluation of multiple aspects of HF patients’ health status with health domains that include physical functioning, symptom frequency and severity, social function, self-efficacy, social interference and overall quality of life [13]. The KCCQ scales are summarized into a single summary score ranging from 0–100, with higher scores reflecting better perceived health status. The PHQ-9 is a clinically validated self-administered 9-item questionnaire for depression [14]. Depression symptoms are stratified and scored as minimal (0–4), mild (5–9), or moderate-to-severe (10–27) with a score ≥10 considered clinically significant for depressive symptoms.

### Study outcomes and follow-up

The primary outcome was defined as 30 days re-hospitalization for ADHF. Outcome adjudication began following study enrollment for Group 1 and after first infusion for Groups 2 and 3. Secondary outcomes included hospitalization beyond 30 days for all cardiac causes, cardiovascular death or myocardial infarction, all-cause death, and changes in KCCQ and PHQ-9 score from baseline to 30 days of follow-up. The 30 days follow-up was obtained through a study clinic visit. After 30 days, outcome ascertainment was obtained through telephone interviews and hospital chart reviews.

### Adverse event monitoring

All episodes of clinical deterioration and adverse events prior to, during, or after the start of the infusion session were documented. Treatment-related hypokalemia or hyponatremia were defined as serum potassium ≤3.5 mEq/l, and serum sodium <130 mEq/l, respectively. Worsening renal function was defined as creatinine level elevation >25% above baseline. Worsening hemodynamics included hypotension (defined as SBP <80 mmHg) or symptoms of presyncope. Other symptoms including dizziness, weakness, ototoxicity, or palpitations following IV medication administration were documented.

### Statistical analysis

For continuous variables, mean and standard deviations were used if the data was normally distributed while median and interquartile ranges were applied for skewed data. For categorical variables, numbers and percentage were used. Baseline characteristics were presented in tabular form for the population as a whole, and in subgroups defined by treatment arm, as well as by HF categories (HFpEF vs HFrEF). The intergroup comparisons were performed by independent t-test or paired t-test for continuous variables and a chi-square test or McNemars test for categorical variables, as deemed appropriate.

Primary outcomes were reported as rates of 30-day hospitalization. Rates of hospitalization and mortality were compared in a subgroup analysis based on HF classification (HFpEF vs HFrEF). Secondary outcomes were reported as beyond 30 days rates of hospitalization for all cardiac causes, cardiovascular death or myocardial infarction, all cause-death; and change in KCCQ and PHQ-9 scores. We report the percentage of patients with a 5-point change in the KCCQ overall summary score, KCCQ overall score <45%, and PHQ-9 score cutoff ≥10, indicating a noticeable clinical difference, and major depressive symptoms, respectively.

Feasibility of outpatient IV diuretic infusion was reported as the percentage of completed sessions. Safety of outpatient IV diuretic therapy in treating HF was reported as the percentage of adverse events occurring during infusion and within 30 days. Resolution of acute HF symptoms was evaluated by NYHA class at baseline infusion visit and at 30 days follow-up [15]. Post-hoc power “per protocol” analysis was conducted based on the observed primary outcome (30 days HF rehospitalization) in the Group 3 (IV furosemide) in comparison to each of the other groups (Group 1: control arm and Group 2: IV placebo infusion). Using two-sided alpha of 0.05 with at least 13% null difference using 2 proportional samples and a power of 90%, a total sample size of 94 was calculated. The study enrolled a total of 100 patients to account for attrition. Intention to treat analysis was also reported for comparing the primary outcome between the 3 groups.

All statistical analyses were performed with JMP Pro 14.1 (SAS Cary, North Carolina). Statistical significance was set *a priori* as two tailed with p <0.05.

## Results

A total of 100 patients consented to the study and were randomized into 3 Groups (Group 1 = 36, Group 2 = 34, and Group 3 = 30). Of those 100 patients initially enrolled, 3 died (2 in Group 2, and 1 in Group 3) after randomization but before the start of the infusion clinic visits, 1 patient (Group 2) withdrew consent from the study, and 2 patients (Group 3) dropped out of the study prior to starting the infusion resulting in a final study population of 94 patients (Group 1 = 36, Group 2 = 31, and Group 3 = 27). (**Fig 1**) Patients in the final study population had a mean age of 64 ± 13 years, including 53 (56%) males, and 65 African Americans. A total of 66 (70%) patients had HFrEF, 89 (94%) had hypertension, and 53 (56%) had diabetes mellitus. A total of 80 (86%) patients were on home loop diuretics with a mean maintenance furosemide diuretic dose of 65 ± 44 mg/day. (**Table 1**) Participants randomized to Group 3 (furosemide infusion), received low dose in 71% of the sessions, and intermediate dosing in 29% of the sessions. Patients with HFpEF were older (mean age 69 ± 12 years) and more likely to be female (74%) compared to those with HFrEF (mean age 62 ± 13 years, 32% female) (**S1 Table**).

**Table 1:** Baseline characteristics categorized by treatment intervention.

Demographics	Overall population (n = 94)	Group 1 (n = 36)	Group 2 (n = 31)	Group 3 (n = 27)
Age (years)	63.8 ± 12.9	62.8 ± 12.2	67 ± 12.9	61.6 ± 13.8
Males, n (%)	53 (56.4%)	23 (63.9%)	15 (48.4%)	15 (55.6%)
Race				
Caucasian	11 (11.7%)	5 (13.9%)	3 (9.7%)	3 (11.1%)
African American	65 (69.1%)	26 (72.2%)	24 (77.4%)	15 (55.6%)
Hispanic	12 (12.8%)	2 (5.6%)	3 (9.7%)	7 (25.9%)
Other	6 (6.2%)	3 (8.3%)	1 (3.2%)	2 (7.4%)
Baseline weight (kg)	95.8 ± 29.5	90.6 ± 26.7	98.9 ± 35.9	99.4 ± 5.7
BMI (kg/m^2^)	32.3 ± 8.8	30.7 ± 9.2	32.1 ± 9.4	34.5 ± 6.9
Obesity (BMI> 30 kg/m^2^)	51 (54.3%)	14 (38.9%)	14 (45.2%)	23 (85.2%)
HFrEF	66 (70.2%)	25 (69.4%)	20 (64.5%)	21 (80.8%)
HFpEF	27 (28.7%)	11 (30.6%)	11 (35.5%)	5 (19.2%)
ICD implantation	29 (31.2%)	11 (30.6%)	12 (38.7%)	6 (23.1%)
CRT	4 (4.3%)	2 (5.6%)	1 (3.2%)	1 (3.9%)
History of atrial fibrillation	30 (31.9%)	13 (36.1%)	9 (29.0%)	8 (29.6%)
Diabetes	53 (56.4%)	19 (52.8%)	21 (67.7%)	13 (48.2%)
Hypercholesterolemia	55 (59.1%)	20 (55.6%)	19 (61.3%)	16 (61.5%)
Hypertension	89 (94.7%)	33 (91.7%)	31 (100%)	25 (92.6%)
Current smoking	13 (14.1%)	3 (8.6%)	6 (20.0%)	4 (14.8%)
Renal disease	41 (51.1%)	15 (41.7%)	19 (61.3%)	14 (51.9%)
History of Cancer	6 (6.5%)	2 (5.6%)	1 (3.2%)	3 (11.5%)
Prior CAD	40 (42.5%)	13 (36.1%)	13 (41.9%)	14 (51.9%)
Prior MI	12 (12.8%)	3 (8.3%)	4 (13.3%)	5 (19.2%)
Prior CABG	12 (12.8%)	5 (13.9%)	3 (9.7%)	4 (14.8%)
COPD	20 (21.3%)	9 (25%)	7 (22.6%)	4 (14.8%)
OSA	17 (18.1%)	4 (11.1%)	8 (25.8%)	5 (18.5%)
PAD	5 (5.3%)	1 (2.8%)	3 (9.7%)	1 (3.7%)
Prior CVA	9 (9.6%)	4 (11.1%)	4 (12.9%)	1 (3.7%)
History of depression	8 (8.6%)	1 (2.9%)	3 (9.7%)	4 (14.8%)
**Cardiac Medications**				
Aspirin	67 (73.6%)	24 (70.6%)	20 (66.7%)	23 (85.2%)
Clopidogrel	12 (13.3%)	6 (17.7%)	2 (6.9%)	4 (14.8%)
Beta blockers	79 (84.9%)	29 (80.6%)	24 (80.0%)	26 (96.3%)
Calcium channel blockers	16 (17.4%)	5 (14.3%)	8 (26.7%)	3 (11.1%)
ACE-I	54 (58.7%)	17 (48.6%)	17 (56.7%)	20 (74.1%)
ARB	15 (16.3%)	7 (20%)	6 (20%)	1 (3.7%)
Loop diuretics: Furosemide	80 (86.0%)	30 (83.3%)	27 (90%)	23 (85.2%)
Other diuretics	23 (25%)	7 (20%)	9 (30%)	7 (25.9%)
Baseline loop diuretic dose, mg/dl	65.3 ± 43.6	67.1 ± 43.1	59.3 ± 28	70.4 ± 58.3
Aldosterone antagonist	31 (33.7%)	11 (31.4%)	8 (26.7%)	12 (44.4%)
Statin	68 (73.9%)	26 (74.3%)	25 (83.3%)	17 (62.9)
Nitrates	24 (26.1%)	8 (22.9%)	8 (26.7%)	8 (29.6%)
Hydralazine	18 (19.6%)	9 (25.7%)	4 (13.3%)	5 (18.5%)
Digoxin	7 (7.4%)	3 (8.3%)	4 (12.9%)	0 (0%)
**Baseline hemodynamics and NYHA class**				
SBP (mmHg)	127.1 ± 21.7	125.4 ± 24.1	125 ± 19.7	131.6 ± 20.5
DBP (mmHg)	73.6 ± 12.4	71.7 ± 11.5	71.3 ± 11.5	78.6 ± 13.5
Heart rate (bpm)	78.9 ± 24.1	80.4 ± 34.1	76 ± 15.1	80.1 ± 15.0
NYHA Classification				
I	0 (0%)	0 (0%)	0 (0%)	0 (0%)
II	12 (12.9%)	6 (17.7%)	3 (10%)	3 (11.1%)
III	25 (26.9%)	13 (38.1%)	8 (26.7%)	4 (14.8%)
IV	54 (58.1%)	15 (44.1%)	19 (63.3%)	20 (74.1%)
**Baseline Labs**				
BUN (mmol/L)	29.8 ± 14	27.2 ± 12.6	32.2 ± 17.0	27.4 ± 11.6
Serum creatinine (mg/dL)	1.27 ± .42	1.19 ± 0.4	1.37 ± 0.5	1.26 ± 0.4
Serum sodium (mmol/L)	139.9 ± 3.1	140 ± 3	139.4 ± 3.6	140 ± 2.5
Serum potassium (mmol/L)	4.27 ± .47	4.2 ± 0.1	4.4 ± 0.1	4.3 ± 0.1
NT-proBNP (pg/ml), median (IQR)	3234 (1755–6154)	4389 (2119–6550)	3227 (1283–5104)	3151 (1796–7294)
**Baseline Echocardiography**				
LVEF, %	33.5 ± 19.3	33.6 ± 18.6	36.2 ± 20.1	30.3 ± 19.7
LVEDd, cm	5.5 ± 1.1	5.6 ± 1.2	5.2 ± 1.1	5.7 ± 0.94
LVESd, cm	4.6 ± 1.4	4.6 ± 1.5	4.5 ± 1.2	4.6 ± 1.6
Stroke Volume, ml	47.6 ± 15.5	45.2 ± 15.1	49.9 ± 16.0	48.3 ± 16.0
Left atrial volume, cc	94.2 ± 34.1	93.2 ± 36.3	94.9 ± 25.1	94.9 ± 41.1
RVSP, mmHg	49 ± 15.3	51.3 ± 15.9	50.1 ± 18.1	45.1 ± 11.3
Mitral E	93.9 ± 40.3	88.0 ± 43.0	102.4 ± 35.3	92.8 ± 41.7
Mitral A	53.1 ± 31.2	48.2 ± 30.4	62.4 ± 29.6	48.9 ± 33.3
E/A	2.28 ± 1.4	2.4 ± 1.7	2.1 ± 1.2	2.3 ± 1.1
e’	7.83 ± 3.2	7.5 ± 2.8	8.2 ± 2.8	7.8 ± 4.2
E/e’	13.7 ± 7.03	12.0 ± 7.6	14.2 ± 6.6	15.2 ± 6.6
Deceleration time, ms	155.6 ± 57.5	154.9 ± 55.4	149.2 ± 57.5	162.3 ± 62.3
IVC diameter, cm	2.08 ± 0.61	2.18 ± 0.60	1.99 ± 0.69	2.06 ± 0.48

Data are expressed as the mean ± SD or number (%) patients or median (Interquartile range). P-values between the three intervention groups obtained from ANOVA test.

ACE-I, angiotensin converting enzyme inhibitor; ARB, angiotensin receptor blocker; BMI, body mass index; BUN, blood urea nitrogen; CABG, coronary artery bypass graft; CAD, coronary artery disease; COPD, chronic obstructive pulmonary disease; CRT, cardiac resynchronization therapy; CVA, cerebrovascular accident; DBP, diastolic blood pressure; HFpEF, heart failure preserved ejection fraction; HFrEF, heart failure reduced ejection fraction; ICD, intracardiac defibrillator; LVEDd, left ventricular end diastolic diameter; LVEF, left ventricular ejection fraction; LVESs, left ventricular end systolic diameter; MI, myocardial infarction; NT-proBNP, N-terminal-pro brain natriuretic peptide; NYHA, New York Heart Association; OSA, obstructive sleep apnea; PAD, peripheral arterial disease; RVSP, right ventricular systolic pressure; SBP, systolic blood pressure.

### Infusion visit metrics

A total of 323 of 464 (69.6%) visits were completed for both Groups 2 and 3. Group 2 completed 167 of 248 (67.3%) planned visits while Group 3 completed 156 of 216 (72.2%) visits. Furosemide diuretics doses in Group 3 were categorized into low (20mg bolus with 20 mg/hr) in 110 of 156 (71%) sessions, intermediate (40 mg bolus with 40 mg/hr) in 46 sessions of 156 (29.5%) sessions and high (80 mg bolus with 80 mg/ hr) in 0 sessions. Overall, infusion sessions were completed as follows: 8 infusion visits in 23 patients, 7 infusion visits in 5 patients, 6 infusion visits in 5 patients, 5 infusion visits in 5 patients, 4 infusion visits in 5 patients, 3 infusion visits in 3 patients, 2 infusion visits in 1 patient, and 1 infusion visit in 9 patients.

**Fig 2:**
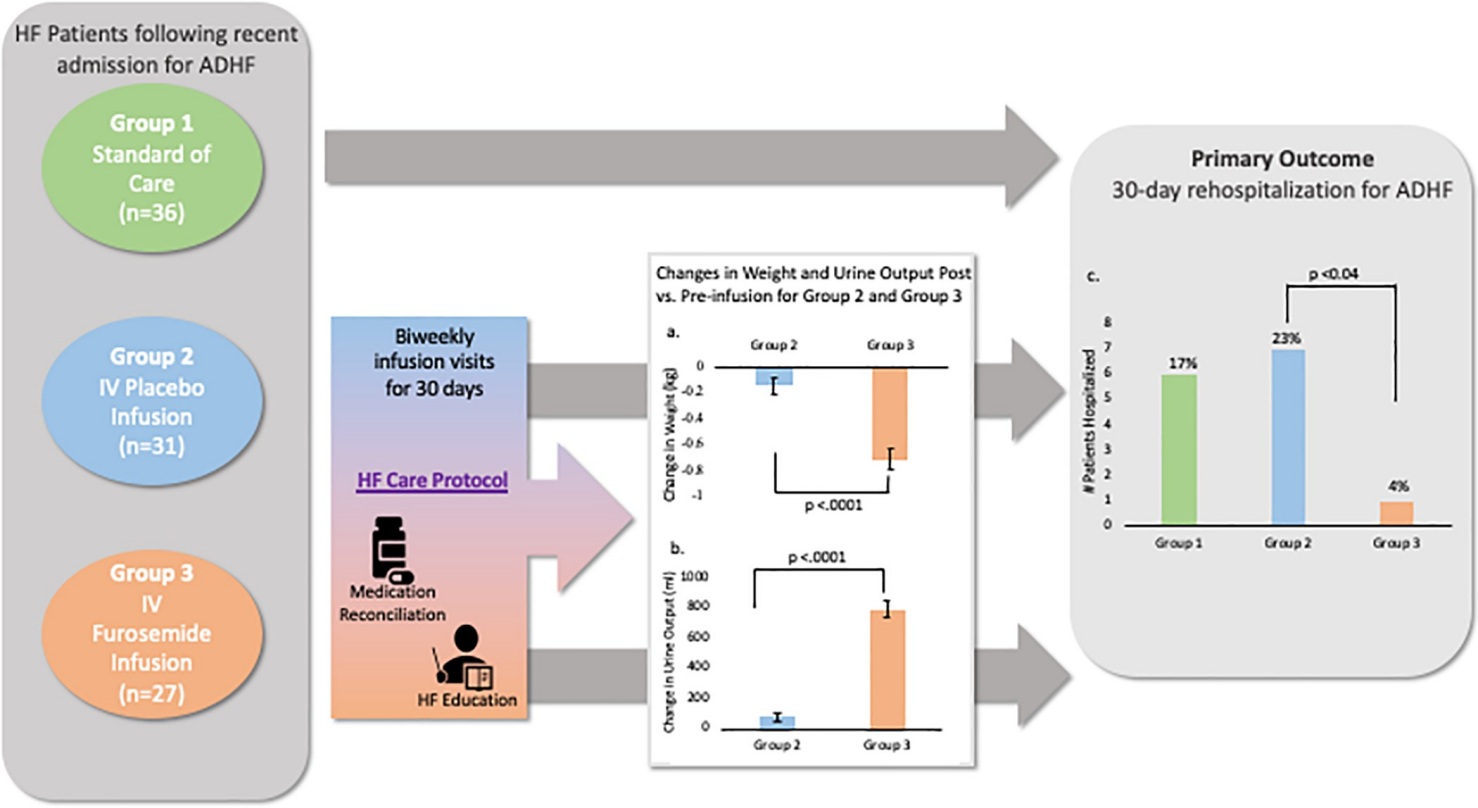
Study outcomes across randomization groups. Patients randomized to Group 1 (standard of care), Group 2 (intravenous placebo infusion), and Group 3 (intravenous furosemide infusion). Groups 2 and 3 underwent biweekly infusion visits for 30 days that included a HF-Care protocol. Changes in weight (a) and urine output (b) post- vs. pre-infusion for Group 2 and Group 3. Primary study outcome results (c) 30-day rehospitalization for ADHF in all three groups. ADHF, acute decompensated heart failure; HF, heart failure; IV, intravenous.

Patients in Group 3 achieved greater weight loss compared to those in Group 2 (mean weight loss of 0.72 kg in Group 3 vs 0.15 kg in Group 2, p <0.0001). Urine output was greater in the Group 3 compared to Group 2 (mean difference of 794.5 ml vs 79.2 ml respectively, p <0.0001). (**Fig 2**) Patients in Group 3 with HFrEF exhibited greater weight loss and urine output compared to those with HFpEF (mean weight loss of 0.79 kg vs 0.45 kg, respectively, p = .04, and mean urine output difference of 861 ml vs 468 ml respectively, p = .0002) (**S2 Table**).

There was a significant difference in SBP (4.8 mmHg) in Group 3 comparing pre- and post-infusion but no other significant hemodynamic differences in Group 3 or Group 2. There was a trend towards NYHA class improvement in Group 3 compared to Group 2. Laboratory values did not change significantly between the 3 groups from baseline to 30-day follow-up, apart from a significant difference in potassium levels and a trend towards significant NT-proBNP reduction in Group 3. (**Table 2**) Echocardiographic parameters such as ejection fraction, end systolic and diastolic volumes as well as diastolic parameters were unchanged from baseline to 30-day follow-up across the 3 groups (**Table 3, S1–S3 Figs**).

**Table 2:** Infusion visit metrics changes (post infusion-pre infusion) categorized by intervention group.

	Group 2	Group 3	*p-value (Between Groups)
	(n = 31)	(n = 27)	
Weight, kg	-0.15 (.07)^¥^	- 0.72 (.08)^¥^	< .0001
Systolic blood pressure, mmHg	-0.85 (1.3)^¥^	- 4.8 (1.4)^¥^	.047
Diastolic blood pressure, mmHg	2.2 (.96) ^¥^	0.52 (.89)	.191
Heart Rate, bpm	0.96 (3.4)	- 0.61 (.64)	.657
Urine output, ml	79.2 (26.2)^¥^	794.5 (52.7)^¥^	< .0001
Serum sodium, mmol/L	-1.29 (0.47) ^¥^	-1.13 (0.29)^¥^	.771
Serum potassium, mmol/L	-0.09 (.06)	-0.14 (.05)^¥^	.518
BUN, mmol/L	-0.86 (0.32)^¥^	- 0.007 (0.3)	.053
Serum creatinine, mg/dL	-0.03 (0.012)^¥^	0.006 (.015)	.109

Data presented as mean difference and standard error (SE) between post-infusion versus pre-infusion values.

*p-values obtained from independent t test or non-parametric test if data is skewed.

^¥^p-value < .05, obtained from student’s paired t-test within each group of intervention (post-infusion vast value–pre-infusion visit value).

BUN, blood urea nitrogen.

**Table 3:** Changes in study outcome at 30 days compared to baseline categorized by treatment intervention group.

	Group 1	Group 2	Group 3	*p-value between groups
	(n = 36)	(n = 31)	(n = 27)	
**Biometrics and Hemodynamics**				
Weight (kg)	-0.28 (1.06)	0.28 (1.05)	-2.5 (1.06) ^¥^	.185
Systolic blood pressure, mmHg	-2.4 (4.7)	7.92 (3.9)	-10.7 (3.5) ^¥^	.017
Diastolic blood pressure, mmHg	0.64 (2.7)	1.56 (2.5)	-9.6 (3.2) ^¥^	.012
Heart rate, bpm	-2.28 (2.5)	3.64 (1.9)	-5.7 (2.9)	.03
**Symptomatology and Questionnaires**				
Change in NYHA Class				
No change	7 (24%)	8 (30.8%)	7 (33.3%)	.934
Improvement by 1 class	11 (37.9%)	11 (42.3%)	7 (33.3%)	
Improvement by 2 classes	2 (6.9%)	3 (11.5%)	3 (14.3%)	
Improvement by 3 classes	2 (6.9%)	3 (11.5%)	3 (14.3%)	
Change in KCCQ scores, median (IQR):				
Total symptom score	14.1 (-5.5–35.4) ^€^	17.7 (-1.3–37.5) ^€^	7.3 (-2.6–38.5) ^€^	.868
Overall summary score	15.4 (3.3–26.9) ^€^	23.9 (11.9–31.01)^€^	17.2 (4.7–35.2) ^€^	.264
Clinical summary score	9.1 (-5.9–19.6) ^€^	10.7 (.78–22.0)^€^	6.3 (-1.04–16.1) ^€^	.424
Change in Overall KCCQ summary score				
Decrease (≥5 point)	3 (10.3%)	1 (4.0%)	1 (4.6%)	.693
No change (<5 point)	4 (13.8%)	2 (8.0%)	4 (18.2%)	
Increase (≥5 point)	22 (75.9%)	22 (88%)	17 (77.3%)	
KCCQ Overall score <45, % Baseline	9 (31%)	8 (30.8%)	7 (33.3%)	.979
Change in PHQ-9, median (IQR)				
Baseline PHQ-9 ≥10	-3.4 (-6.5–0) ^€^	-0.5 (-2.5–1.5)	-2.5 (-7.5–0) ^€^	.291
30-day PHQ-9 ≥10	13 (36.1%)	8 (25.8%)	11 (42.3%)	.410
	4 (13.3%)	5 (19.2%)	4 (19.1%)	.802
**Labs**				
Serum BUN, mmol/L	-0.94 (2.5)	-0.96 (2.5)	3.4 (3.3)	.376
Serum creatinine, mg/ld.	0.21 (0.09) ^¥^	.04 (.08)	0.15 (.06) ^¥^	.333
Serum sodium, mmol/L	-0.703 (0.66)	-2.04 (.65)^¥^	-0.77 (0.7)	.248
Serum potassium, mmol/L	0.27 (0.12) ^¥^	-0.23 (0.12)	.02 (0.12)	.014
NT-proBNP, pg/ml	-2586.3 (822) ^¥^	2416.5 (3481.3)	-5253.3 (3043.5)	.152
**Echocardiography**				
LVEF, %	2.8 (1.7)	2.05 (1.2)	2.0 (2.6)	.931
LVEDd, cm	.37 (.2)	0.45(.23)	0.35 (.25)	.945
LVESd, cm	.34 (.15)	.04(.17)	0.37 (.18)	.246
Stroke Volume, ml	12.6 (5.1)	4.9(6.5)	11.8 (6.9)	.644
Left atrial volume, cc	-.58 (5.8)	-9.8(5.4)	1.5 (8.2)	.344
RVSP, mmHg	-9.2 (3.1)	-8.3 (3.4)	-4.5 (3.8)	.575
E/e’	3.7 (2.1)	0.82 (2.2)	-.8 (2.4)	.112
IVC diameter, cm	0.38 (0.13)	0.17 (0.15)	0.05 (0.12)	.315
**Events at 30 days**				
Re hospitalization for HF	6 (17.1%)	7 (22.6%)^#^	1 (3.7%)^#^	.107
Cardiac hospitalization for non- HF causes	4 (11.8%)	2 (6.7%)	1 (3.7)	.619

Data presented as mean difference and standard error (SE) between baseline versus 30-day values.

*p-values between the three intervention groups obtained from ANOVA test.

^¥^ p-values < .05, obtained from student’s paired t-test within each group (30 days follow-up value-baseline value).

^€^p-values < .05, obtained from Wilcoxon signed rank test paired t-test for non-parametric skewed data within each group (30 days follow-up value—baseline value).

^#^Comparing 30-day hospitalization between group 2 and group 3, p = .037.

BUN, blood urea nitrogen; HF, heart failure; KCCQ, Kansas City Cardiomyopathy Questionnaire; LVEDd, left ventricular end diastolic diameter; LVEF, left ventricular ejection fraction; LVESs, left ventricular end systolic diameter; NT-proBNP, N-terminal-pro brain natriuretic peptide; NYHA, New York Heart Association; PHQ-9, Patient Health Questionnaire-9; RVSP, right ventricular systolic pressure.

Adverse events before or during the start of infusion were reported in 28/464 (6%) visits with 17 events in Group 2 and 11 events in Group 3. These included hypotension in 2 visits, increase in serum creatinine in 9 visits, hypokalemia in 6 visits, hypomagnesemia in 5 visits, hyperkalemia in 2 visits, symptomatology of chest pain in 1, shortness of breath in 1, and runs of non-sustained ventricular tachycardia in 2 visits.

### 30-day follow-up results

Out of 94 patients, 93 (99%) completed the 30 days follow-up clinic visit. There were a total of 14 (15%) hospitalizations for ADHF at 30 days, 6 (17.1%) in Group 1, 7 (22.6%) in Group 2, and 1 (3.7%) in Group 3 (p = 0.037 comparing Group 2 and Group 3). (**Fig 2**) Similarly, the intention to treat analysis showed a total of 15 (15%) hospitalizations for ADHF at 30 days, 6 (17.1%) in Group 1, 8 (23.5%) in Group 2, and 1 (3.7%) in Group 3 (p = 0.020 comparing Group 2 and Group 3).

At 30 days follow-up, there were no cardiac or non-cardiac deaths. A total of 7 (8%) patients experienced 30 days hospitalization for causes other than HF (uncontrolled hypertension in 2 patients (Group 1), chest pain in 2 patients (Group 2), scalp abscess in 1 patient (Group 1), ICD shock for polymorphic VT in 1 patient (Group 3), and bowel obstruction for 1 patient (Group 1)). (**Table 3**) There were no significant differences in outcomes among participants with HFrEF versus HFpEF (**S3 Table**).

### Beyond 30-day follow-up results

Beyond 30-day follow-up was available in 90 patients **(**2.8 ± 2.2 years). At 180 days of follow-up, hospitalizations for ADHF were reported in a total of 31 (34.4%) patients, 11 (36.7%) in Group 1, 12 (42.9%) in Group 2, and 7 (30.8%) in Group 3. Hospitalization for causes other than HF was reported in 14 (16.4%) patients. Of those, 1 patient was hospitalized for hypotension, 4 patients with cerebrovascular accident (CVA)/transient ischemic attack (TIA), 2 patients with chest pain/acute coronary syndrome (ACS), 1 patient with syncope, and the remainder were hospitalized for non-cardiac causes.

Beyond 180 days of follow-up, hospitalizations for ADHF were reported in 44 (48.9%) patients, 14 (50%) in Group 1, 16 (57%) in Group 2, and 14 (56%) in Group 3. Hospitalization for causes other than HF were reported in 43 (53%) patients. Of those, 1 patient was hospitalized for hypertensive emergency, 2 for ICD implantation, 1 with chest pain/ACS, 2 patients with hypotension/syncope, and the remainder for non-cardiac causes.

All cause-mortality during the study follow-up period beyond the 30 days occurred in 16 (17.7%) patients (5 (13.8%) in Group 1, 8 (25.8%) in Group 2, and 3 (11.1%) in Group 3 (overall p = 0.224, p = 0.11 between Group 2 and 3). Of those, 10 patients (1 (2.7%) in Group 1, 6 (19.4%) in Group 2, and 3 (11.1%) in Group 3) experienced a cardiac cause of death related to severe ADHF or cardiogenic shock.

### KCCQ and PHQ-9 results

A total of 77 (82%) patients had completed KCCQ data at baseline and 30-day follow-up. The median KCCQ overall summary score in all groups was 38.5 (IQR 24.2–53.7) at baseline, and 65.6 (IQR 43.8–81.3) at 30 days follow-up. From baseline to 30 days follow-up, 61 patients (80.3%) experienced a ≥5 point improvement in health status, while 5 patients (6.5%) experienced a ≥5 point decline. A higher proportion of patients who experienced ≥5 point improvement in KCCQ overall summary score were characterized as having NYHA class IV symptom limitations at baseline compared to those who reported a ≥5 point decline (57.6% vs. 40%, p = .004). There was a statistically significant change within groups with respect to KCCQ total symptom score, overall summary score, and clinical summary score however there were no significant between group differences (**Table 3**).

A total of 77 patients completed the baseline and 30 days follow-up PHQ-9 questionnaire. There was a statistically significant change within groups with respect to PHQ-9 total score. However, no significant changes were observed in between-group comparisons (**Table 3**).

## Discussion

In this randomized double blind placebo-controlled trial of 94 adult men and women following hospitalization for ADHF, we found that treatment following hospital discharge in an ambulatory diuretic infusion clinic with IV furosemide twice weekly for one month was associated with a significant reduction in the frequency of rehospitalization for ADHF at 30 days follow-up (3.7%) compared to placebo infusion and standard of care (22.6% and 17.1%, respectively). In addition, we found no documented adverse events with the use of IV diuretics. To our knowledge, our study is one of the first randomized controlled double blind studies evaluating the role of outpatient IV diuretic infusion clinics with a multidisciplinary approach to the treatment of HF to reduce 30 days re-admission for ADHF. Our overall rate of HF rehospitalization was reduced compared to the reported rates of 20–25% [16].

We adopted the approach used in the Diuretic Optimization Strategies Evaluation (DOSE) trial and utilized a standardized furosemide infusion protocol which consisted of a furosemide bolus followed by a 3-hour infusion [17]. Our study showed as expected, a significant increase in urine output and weight loss in the IV furosemide group compared to the other two intervention groups. We found no significant differences in hemodynamic parameters including blood pressure or laboratory parameters in placebo versus furosemide infusion groups. Among patients receiving IV furosemide, patients with HFrEF experienced significant weight loss and increased urine output compared to those with HFpEF. Nonetheless the rate of worsening renal function (increased BUN or creatinine levels), were not different across both HF subtypes.

Our results extend prior observational cohort studies evaluating the role of IV diuretics in ambulatory settings, supporting the reduction of HF re-hospitalization [10, 11, 18–23]. In a study of 60 chronic HF patients receiving outpatient IV furosemide bolus followed by 3-hour infusion, investigators found that infusions were associated with a median urine output of 1.1 L and 24 hours weight loss of 1.1 kg for the entire cohort including patients with HFrEF and HFpEF with an observed rate of all-cause hospitalization at 30 days of 31.7%, with no deaths.20 We report a similarly significant weight loss and urine output albeit not to the same degree. The differences may be due to heterogeneity of the baseline home diuretic dose (240mg daily furosemide home dose) compared to our study (70 mg daily furosemide home dose). Our study adds further to previous studies with the strength and uniqueness of its methodology as a randomized controlled trial, enrollment of both HFrEF and HFpEF patients, with a large representation of comorbidities, detailed monitoring of patients during infusions, and a longer duration of follow-up.

Despite significant within group comparisons in KCCQ and PHQ-9 scores, we were not able to detect significant between-group changes. This may be due to the smaller proportion of patients experiencing a large magnitude of change in the questionnaire scores which may have limited the power to detect associations between improvements in the scores and outcome.

This analysis has several limitations. Our study included a modest sample size from a single center. Notably, we recruited patients from a large urban center with a diverse population including 69% African Americans. Our analysis lacks reporting on hospital length of stay. Our study included unbalanced group sizes, which can be attributed to the differences in recruitment rate, a higher than expected loss to follow-up, time-research personnel logistics and budget constraints. However, the power of the study was maintained above 90% for both “per protocol” and “intension to treat” analyses. In our study design the standard of care group monitoring was solely an observatory arm and management was at the discretion of the HF specialty clinic. We acknowledge that some variations among cardiology practices between patient treatment and published evidence-based HF guidelines exist which may have influenced outcomes in the study. Given the large discrepancy in urine output between groups receiving placebo infusion (Group 2) and furosemide infusion (Group 3), it is possible that study personnel may have been able to determine randomization allocation, limiting blinding. Furthermore, a cost analysis of bi-weekly outpatient diuretic infusion is important however beyond the scope of the study design.

Future clinical approaches to patient care are in line with evidence-based strategies utilizing a multidisciplinary care team in tailoring HF management. These evidence based strategies include the implementation of dedicated ambulatory outpatient monitoring clinics (including monitoring of hemodynamic data, weight, volume status, medication adherence, and salt intake) coupled with intervention (where IV diuretics are administered on an as-need basis). This approach may ultimately facilitate the decentralization of readmissions to hospitals, decreasing the healthcare cost burden and worsening outcomes in patients with ADHF.

## Conclusions

The ambulatory management of hemodynamically stable patients with ADHF, including those with HFrEF and HFpEF, utilizing a standardized protocol with IV diuretic treatment is feasible, safe, and effective in reducing 30 days re-hospitalization.

## Supporting information

S1 Fig Baseline and follow-up echo images from patient in standard of care group. Apical 4-Chamber View at Baseline (a) Parasternal Short Axis View at Baseline (b) Apical 4-Chamber View at Follow-up (c) Parasternal Short Axis View at Follow-up (d).
(TIFF)

S2 Fig Pre- and post-infusion echo images from patient in IV placebo group. Apical 4-Chamber View Pre-infusion (a) Parasternal Short Axis View Pre-infusion (b) Apical 4-Chamber View Post-infusion (c) Parasternal Short Axis View Post-infusion (d).
(TIFF)

S3 Fig Pre- and post-infusion echo images from patient in IV furosemide group. Apical 4-Chamber View Pre-infusion (a) Parasternal Short Axis View Pre-infusion (b) Apical 4-Chamber View Post-infusion (c) Parasternal Short Axis View Post-infusion (d).
(TIFF)

S1 Table Baseline characteristics categorized by HF type. (DOCX)

S2 Table Infusion visit metrics changes (post infusion-pre infusion) categorized by intervention group and HF type. (DOCX)

S3 Table Changes in study outcome at 30 Days compared to baseline categorized by HF type. (DOCX)

S1 File OUTLAST study protocol. (DOC)
