# Supplementary figures and images for: OUTpatient intravenous LASix Trial in reducing hospitalization for acute decompensated heart failure (OUTLAST)
Source: PLoS One. 2021 Jun 25;16(6):e0253014. doi: 10.1371/journal.pone.0253014 (PMC8232441; doi:10.1371/journal.pone.0253014)

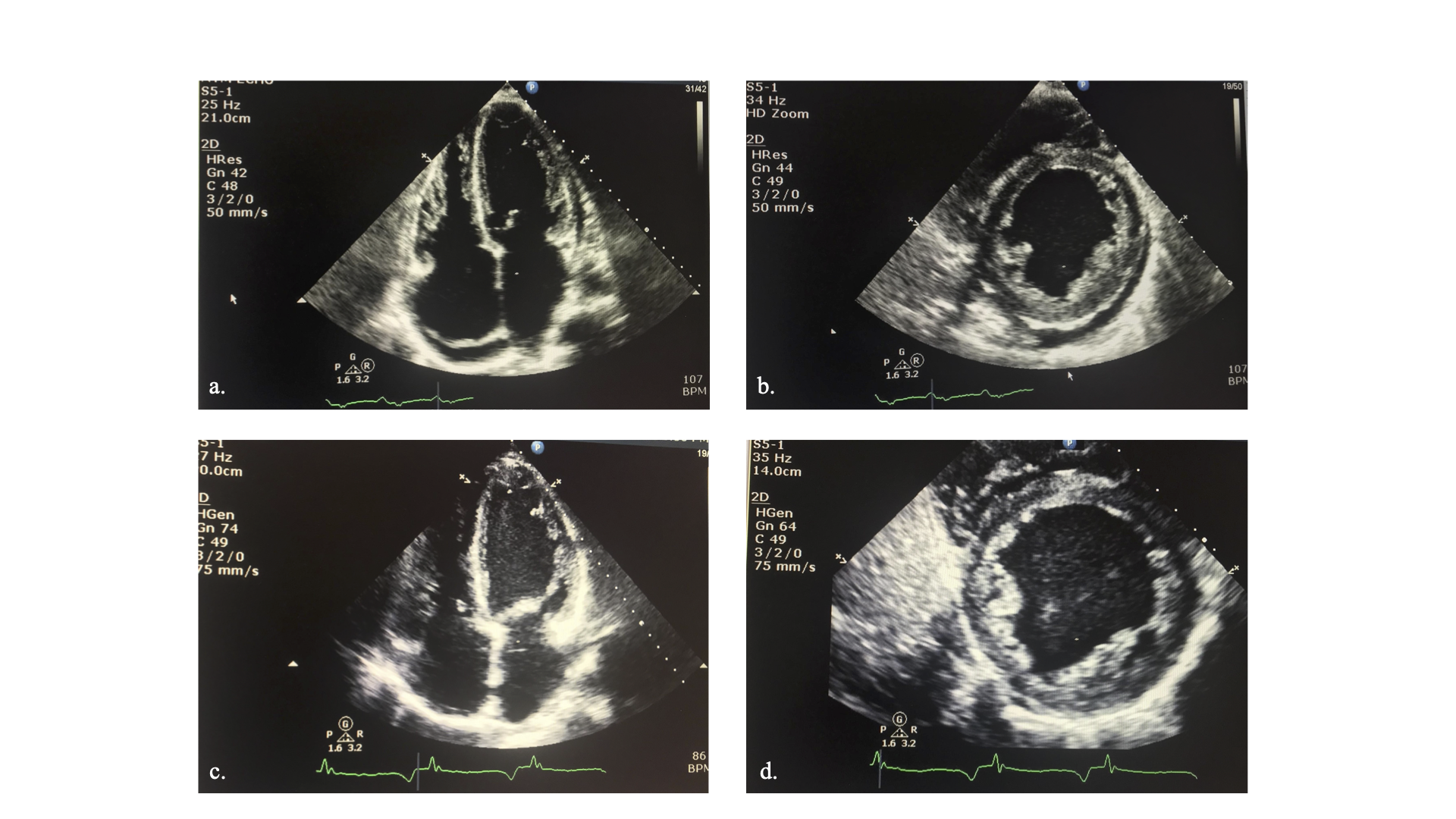

Supplement: S1 Fig — Apical 4-Chamber View at Baseline (a) Parasternal Short Axis View at Baseline (b) Apical 4-Chamber View at Follow-up (c) Parasternal Short Axis View at Follow-up (d). (TIFF) [file pone.0253014.s001.tiff]

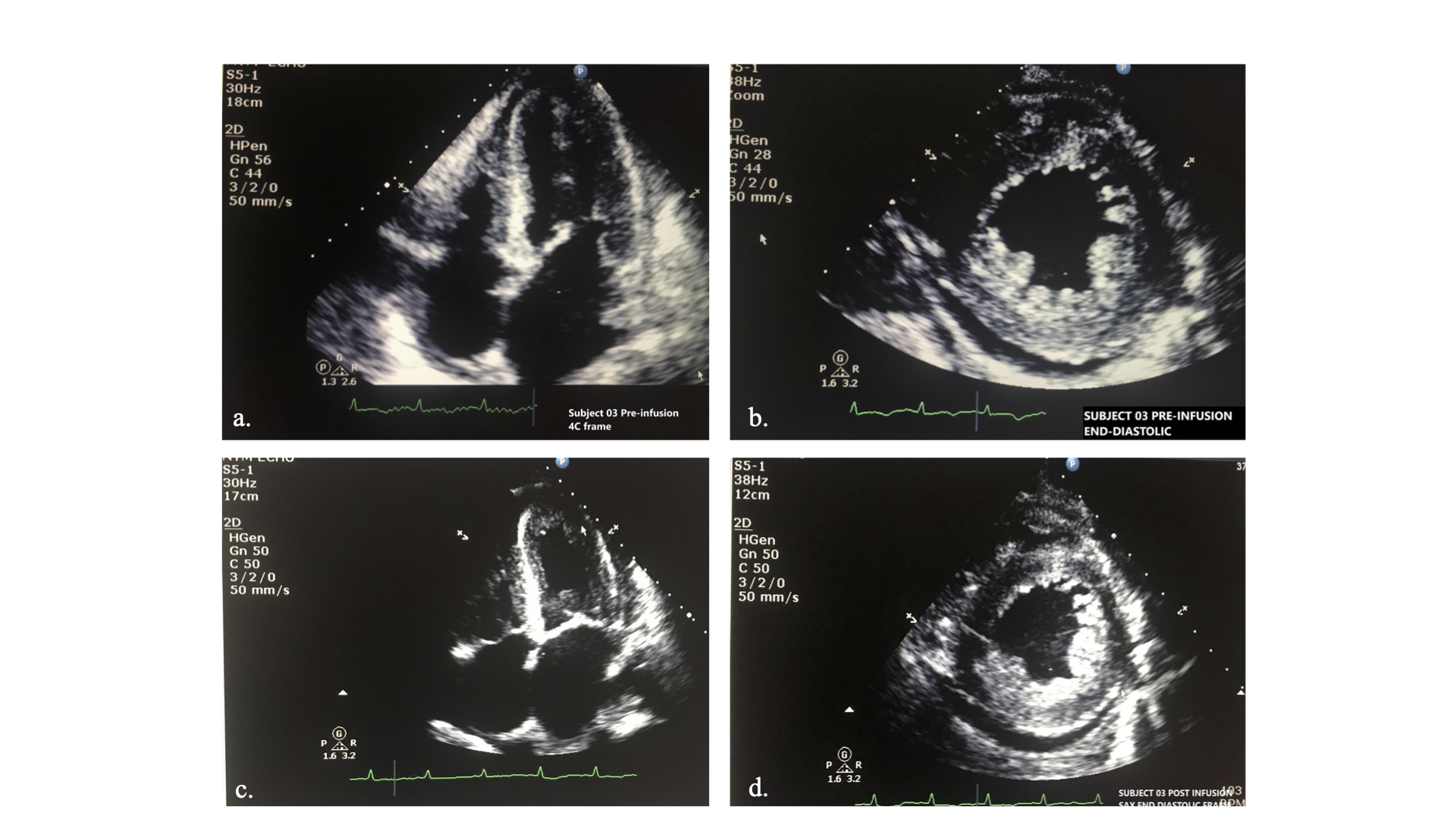

Supplement: S2 Fig — Apical 4-Chamber View Pre-infusion (a) Parasternal Short Axis View Pre-infusion (b) Apical 4-Chamber View Post-infusion (c) Parasternal Short Axis View Post-infusion (d). (TIFF) [file pone.0253014.s002.tiff]

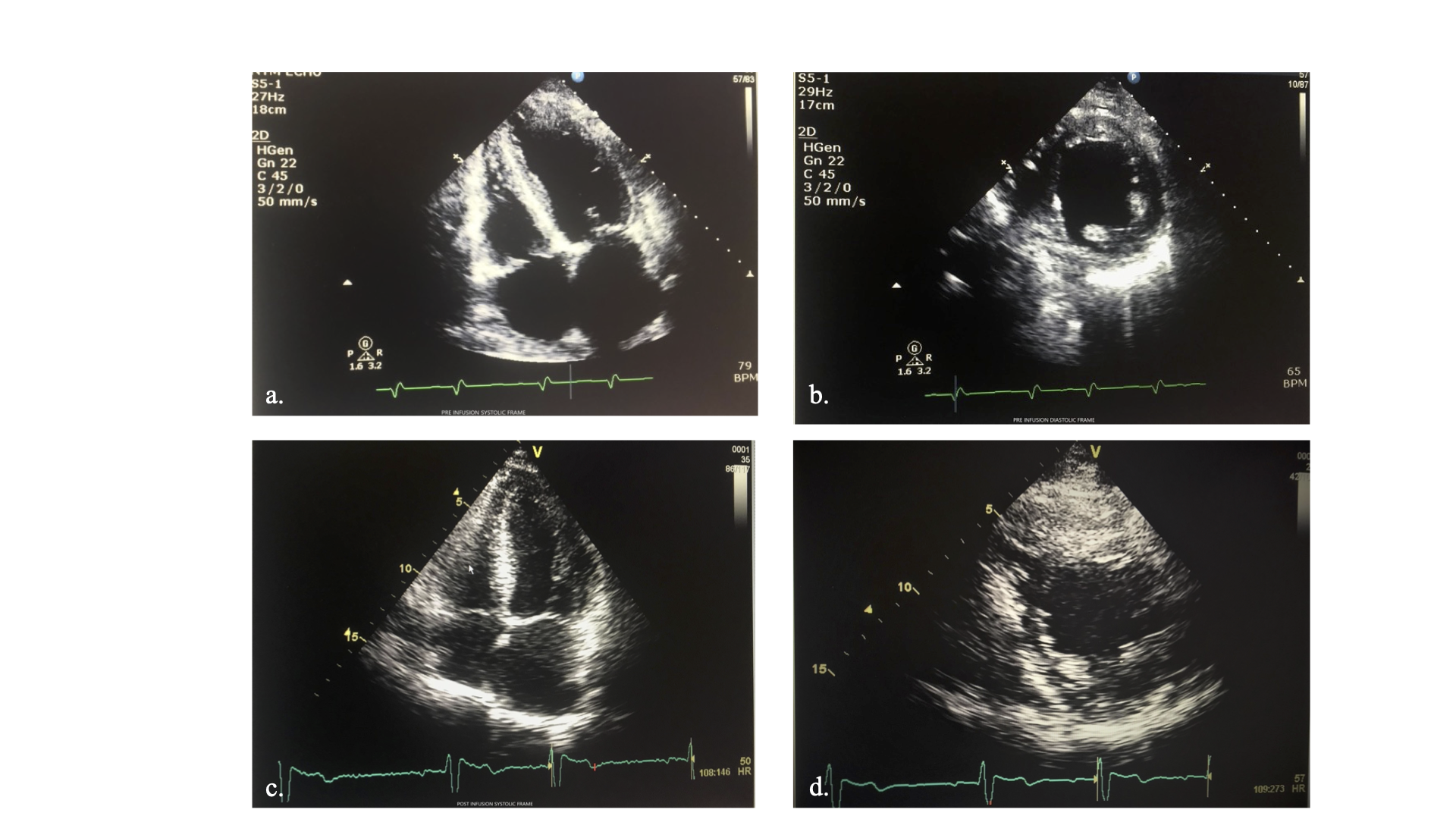

Supplement: S3 Fig — Apical 4-Chamber View Pre-infusion (a) Parasternal Short Axis View Pre-infusion (b) Apical 4-Chamber View Post-infusion (c) Parasternal Short Axis View Post-infusion (d). (TIFF) [file pone.0253014.s003.tiff]
