# Supplementary material for: OUTpatient intravenous LASix Trial in reducing hospitalization for acute decompensated heart failure (OUTLAST)
Source: PLoS One. 2021 Jun 25;16(6):e0253014. doi: 10.1371/journal.pone.0253014 (PMC8232441; doi:10.1371/journal.pone.0253014)
Supplement: S1 Table — (DOCX) [file pone.0253014.s004.docx]

**S1 Table.** Baseline Characteristics Categorized by HF Type

| **Demographics** | **HFrEF**  **(n=66)** | **HFpEF**  **(n=27)** | **p-value** |
| --- | --- | --- | --- |
| Treatment arm  Group 1 (Standard care)  Group 2 (Placebo)  Group 3 (Lasix) | 25 (37.9%)  20 (30.3%)  21 (31.8%) | 11 (40.7%)  11 (40.7%)  5 (18.5%) | .391 |
| Age, years | 61.5 ± 12.8 | 69.3 ± 12 | .009 |
| Males, n (%) | 45 (68.2%) | 7 (25.9%) | .0002 |
| Race  Caucasian  African American  Hispanic  Other | 8 (12.1%)  42 (63.6%)  10 (15%)  6 (9%) | 3 (11.1%)  22 (81.5%)  2 (7.4%)  0 (0%) | .373 |
| Baseline weight, kg | 93.2 ± 31.0 | 95.4 ± 26.6 | .893 |
| BMI, kg/m^2^ | 31.7 ± 8.9 | 33.8 ± 9.1 | .318 |
| Obesity (BMI > 30 kg/m^2^) | 32 (48.5%) | 18 (66.7%) | .110 |
| ICD implantation | 25 (38.5%) | 4 (14.8%) | .074 |
| CRT | 3 (4.6%) | 1 (3.7%) | .073 |
| History of atrial fibrillation | 22 (33.3%) | 8 (29.6%) | .787 |
| Diabetes | 35 (53%) | 18 (66.7%) | .228 |
| Hypercholesterolemia | 38 (58.9%) | 17 (62.9%) | .688 |
| Hypertension | 61 (92.4%) | 27 (100%) | .141 |
| Current smoking | 11 (17.2%) | 2 (7.4%) | .414 |
| Renal disease | 30 (45.5%) | 18 (66.7%) | .063 |
| History of Cancer | 5 (7.7%) | 1 (3.7%) | .481 |
| Prior CAD | 32 (48.5%) | 8 (29.6%) | .096 |
| Prior MI | 8 (12.5%) | 4 (14.8%) | .766 |
| Prior CABG | 9 (13.6%) | 3 (11.1%) | .742 |
| COPD | 12 (18.2%) | 8 (29.6%) | .223 |
| OSA | 10 (15.2%) | 7 (25.9%) | .222 |
| PAD | 4 (6.1%) | 1 (3.7%) | .647 |
| Prior CVA | 6 (9.1%) | 3 (11.5%) | .765 |
| History of depression | 4 (6.1%) | 4 (15.4%) | .153 |
| **Cardiac Medications** |  |  |  |
| Aspirin | 47 (72.3%) | 19 (76%) | .725 |
| Clopidogrel | 6 (9.5%) | 6 (23.1%) | .089 |
| Beta blockers | 56 (86.2%) | 22 (81.5%) | .569 |
| Calcium channel blockers | 2 (3.3%) | 13 (50%) | <.0001 |
| ACE-I | 44 (67.7%) | 9 (34.6%) | .009 |
| ARB | 5 (7.7%) | 10 (38.5%) | .0004 |
| Loop diuretics: Furosemide | 55 (84.5%) | 24 (88.9%) | .592 |
| Other diuretics | 16 (24.6%) | 7 (26.9%) | .819 |
| Baseline loop diuretic dose, mg/dl | 53.3 ± 21.8 | 70.4 ± 5.6 | .029 |
| Aldosterone antagonist | 27 (41.5%) | 4 (15.4%) | .017 |
| Statin | 45 (69.2%) | 22 (84.6%) | .132 |
| Nitrates | 20 (308%) | 4 (15.4%) | .118 |
| Hydralazine | 16 (24.6%) | 2 (8%) | .067 |
| Digoxin | 6 (9.1%) | 1 (3.7%) | .246 |
| **Baseline hemodynamics and NYHA class** |  |  |  |
| SBP, mmHg | 135.1 ± 20.4 | 123.7 ± 21.6 | .021 |
| DBP, mmHg | 72.2 ± 9.2 | 73.7 ± 13.2 | .550 |
| Hear rate, bpm | 81.3 ± 39.6 | 77.9 ± 14.8 | .676 |
| NYHA Class  I  II  III  IV | 0 (0%)  11 (16.7%)  16 (24.2%)  37 (56.1%) | 0 (0%)  1 (3.9%)  9 (34.6%)  16 (61.5%) | .204 |
| **Baseline Labs** |  |  |  |
| BUN | 34.5 ± 16.4 | 26.9 ± 12.4 | .016 |
| Serum creatinine | 1.29 ± 0.5 | 1.5 ± 0.5 | .069 |
| Serum sodium, mmol/L | 139.8 ± 2.8 | 139.9 ± 3.8 | .960 |
| Serum potassium, mmol/L | 4.3 ± 0.5 | 4.3 ± 0.5 | .966 |
| NT-pro-BNP, pg/ml, median (IQR) | 2899  (1481-4683) | 3858  (1870-7106) | .115 |
| **Baseline Echocardiography** |  |  |  |
| LVEDd, cm | 5.9 ± 1.05 | 4.6 ± 0.68 | <.0001 |
| LVESd, cm | 5.2 ± 1.3 | 3.2 ± 0.56 | <.0001 |
| Stroke Volume, ml | 42.8 ± 13.6 | 60.8 ± 12.9 | .0002 |
| Left atrial volume, cc | 100.9 ± 32.6 | 78.0 ± 32.8 | .004 |
| RVSP, mmHg | 49.5 ± 14.9 | 48.2 ± 16.7 | .793 |
| Mitral E | 91.8 ± 39.5 | 99.3 ± 42.3 | .442 |
| Mitral A | 46.8 ± 26.7 | 69.2 ± 36.6 | .014 |
| E/A | 2.5 ± 1.4 | 1.8 ± 1.3 | .041 |
| e’ | 7.5 ± 3.5 | 8.6 ± 2.6 | 0.113 |
| E/e’ | 14.4 ± 7.1 | 12.1 ± 6.8 | 0.188 |
| Data are expressed as the mean ± SD or number (%) patients or median (Interquartile range). P-value obtained from intergroup comparisons were performed by independent t-test for continuous variables and a chi-square test for categorical variables, as deemed appropriate.  ACE-I, angiotensin converting enzyme inhibitors; ARB angiotensin receptor blocker; BMI: body mass index; BUN, blood urea nitrogen; CABG, coronary artery bypass graft; CAD: coronary artery disease; CRT, cardiac resynchronization therapy; CVA, cerebrovascular accident; DBP, diastolic blood pressure; HFpEF, heart failure preserved ejection fraction; HFrEF, heart failure reduced ejection fraction; HTN, hypertension; ICD, implantable cardiac defibrillator; LVEDd, left ventricular end diastolic diameter; LVEF, left ventricular ejection fraction; LVESs, left ventricular end systolic diameter; MI, myocardial infarction; NT-proBNP, N-terminal-pro brain natriuretic peptide; NYHA, New York Heart Association; OSA, obstructive sleep apnea; PAD, peripheral arterial disease; PCI, percutaneous intervention; RVSP, right ventricular systolic pressure; SBP, systolic blood pressure. | | | |
