# Supplementary material for: OUTpatient intravenous LASix Trial in reducing hospitalization for acute decompensated heart failure (OUTLAST)
Source: PLoS One. 2021 Jun 25;16(6):e0253014. doi: 10.1371/journal.pone.0253014 (PMC8232441; doi:10.1371/journal.pone.0253014)
Supplement: S2 Table — (DOCX) [file pone.0253014.s005.docx]

**S2 Table.** Infusion Visit Metrics Changes (post infusion-pre infusion) Categorized by Intervention Group and HF Type

| **Group 2** | **HFrEF (n=20)** | **HFpEF (n=11)** | ***p-value between groups** |
| --- | --- | --- | --- |
| Weight, kg | -0.09 (.09) | -0.31 (.15)^¥^ | .196 |
| Systolic blood pressure, mmHg | - 0.38 (1.6) | -1.9 (2.8) | .609 |
| Diastolic blood pressure, mmHg | 2.9 (1.2)^¥^ | 0.79 (1.4) | .346 |
| Heart Rate, bpm | 3.1(4.7) | -4.4 (1.6)^¥^ | .329 |
| Urine output, ml | 105.3 (29.9)^¥^ | 20.4 (54.2) | .126 |
| Serum sodium, mmol/L | -1.58 (0.64)^¥^ | -0.6 (0.42) | .358 |
| Serum potassium, mmol/L | -0.07 (.07) | -0.14(.13) | .590 |
| BUN, mmol/L | - 0.93 (0.4)^¥^ | -0.7 (0.4) | .747 |
| Serum creatinine, mg/dL | -0.04 (.02)^¥^ | -0.02 (0.02) | .025 |
| **Group 3** | **HFrEF (n=21)** | **HFpEF (n=5)** | ***p-value between groups** |
| Weight, kg | -0.79 (.09)^¥^ | -0.45 (.14)^¥^ | .044 |
| Systolic blood pressure, mmHg | - 4.1 (1.7)^¥^ | -8.6 (2.5)^¥^ | .146 |
| Diastolic blood pressure, mmHg | 1.09 (1.02) | -1.9 (2.1) | .206 |
| Heart rate, bpm | -0.44 (.77) | -1.5 (1.3) | .482 |
| Urine output, ml | 860.9 (64.3)^¥^ | 467.9 (68.9)^¥^ | .0002 |
| Serum sodium, mmol/L | -1.27 (0.36)^¥^ | -1.26 (.58)^¥^ | .989 |
| Serum potassium, mmol/L | -0.11 (.06) | -0.16 (.10) | .674 |
| BUN, mmol/L | - 0.09 (0.38) | -0.03 (0.57) | .935 |
| Serum creatinine, mg/dL | .01 (.02) | -0.02 (0.02) | .892 |
| Data presented as mean difference and standard error (SE) between post-infusion versus pre-infusion values.  *p-values obtained from independent t test or non-parametric test if data is skewed.  ^¥^p-values <.05, obtained from student’s paired t-test within each group of HF (post infusion visit value – pre-infusion visit value)  BUN, blood urea nitrogen; HFpEF, heart failure preserved ejection fraction; HFrEF, heart failure reduced ejection fraction. | | | |
