# Supplementary material for: OUTpatient intravenous LASix Trial in reducing hospitalization for acute decompensated heart failure (OUTLAST)
Source: PLoS One. 2021 Jun 25;16(6):e0253014. doi: 10.1371/journal.pone.0253014 (PMC8232441; doi:10.1371/journal.pone.0253014)
Supplement: S3 Table — (DOCX) [file pone.0253014.s006.docx]

**S3 Table.** Changes in Study Outcome at 30 Days Compared to Baseline Categorized by HF Type

|  | **HFrEF** | **HFpEF** | ***p-value between three groups** |
| --- | --- | --- | --- |
| **Biometrics and Hemodynamics** | | | |
| Weight, kg | -2.72 (1.2) | -1.75 (2.5) | .813 |
| Systolic blood pressure, mmHg | -10.9 (4.2) | 11 (8.3) | .670 |
| Diastolic blood pressure, mmHg | -9.4 (3.7) | -7.3 (7.5) | .776 |
| Heart Rate, bpm | -6.6 (3.5) | -1.5 (6.9) | .634 |
| **Symptomatology and Questionnaires** | | | |
| Change in NYHA class  No change  Improvement by 1 class | 9 (60%)  6 (40%) | 2 (66.7%)  1 (33.3%) | .828 |
| Change in Overall KCCQ Summary Score, median (IQR) | 16.6 (6.5-32.5) | 19.8 (7.1-28.3) | .683 |
| Change in PHQ-9 Score, median (IQR) | -1.0 (-7.5-0) | -2 (-4-0) | .540 |
| **Labs** | | | |
| Serum BUN, mmol/L | 3.4 (3.9) | 4.0 (8.2) | .949 |
| Serum creatinine, mg/dL | 0.08 (0.06) | 0.44 (.28) | .041 |
| Serum sodium, mmol/L | -1.1 (0.66) | -0.77 (0.7) | .241 |
| Serum potassium, mmol/L | 0.11 (0.14) | -.23 (.28) | .293 |
| pBNP, pg/ml | -7102 (3651) | -99.5 (6830) | .313 |
| **Echocardiography** | | | |
| LVEDd, cm | .34 (.3) | 0.42 (.26) | .795 |
| LVESd, cm | .35 (.13) | 0.51 (.3) | .695 |
| Stroke Volume, ml | 12.6 (5.1) | 11.8 (6.9) | .635 |
| Left atrial volume, cc | -.05 (9.6) | -11.1 (21) | .344 |
| RVSP, mmHg | -5.4 (4.7) | 6 (15) | .429 |
| E/e’ | -.95 (1.6) | -.43 (3.8) | .901 |
| **Events at 30 days** | | | |
| 30 days re-hospitalization for HF | 1 (4.8%) | 0 (0%) | .619 |
| Data presented as mean difference and standard error (SE) between baseline versus 30-day values.  *p-values between the three intervention groups obtained from ANOVA test  HF, heart failure; HFpEF, heart failure preserved ejection fraction; HFrEF, heart failure reduced ejection fraction; KCCQ, Kansas City Cardiomyopathy Questionnaire; LVEDd, left ventricular end diastolic diameter; LVESs, left ventricular end systolic diameter; NT-proBNP, N-terminal-pro brain natriuretic peptide; NYHA, New York Heart Association; PHQ-9, Patient Health Questionnaire-9; RVSP, right ventricular systolic pressure. | | | |
