# Supplementary material for: OUTpatient intravenous LASix Trial in reducing hospitalization for acute decompensated heart failure (OUTLAST)
Source: PLoS One. 2021 Jun 25;16(6):e0253014. doi: 10.1371/journal.pone.0253014 (PMC8232441; doi:10.1371/journal.pone.0253014)
Supplement: S1 File — (DOC) [file pone.0253014.s007.doc]

**OUTpatient Intravenous LASix Trial in Reducing Hospitalization for ADHF (OUTLAST)**

**I. BACKGROUND**

Heart failure (HF) is a chronic health condition affecting 1-2% of the population (McMurray 2000) characterized by high mortality and frequent episodes of decompensation requiring hospitalizations (Jencks 2009) (Roger 2011). According to Medicare claims, 21% of the beneficiaries are rehospitalized in 30 days, with HF being the most common reason (Jencks 2009). Rates of readmission for HF has been reported to be from 10% to 19% at 2 weeks (Ashton 1995) to as high as 50% within 3 months (Vinson 1990).

Increase in prevalence of HF (van Jaarsveld 2006)(Roger 2011) coupled with extended, frequent hospital stays represents an enormous burden to our healthcare system. The estimated U.S. inpatient hospital charges in 2006 for HF was $32.7 billion (Andrews 2008) and is one of the top 5 most expensive conditions.

Many HF admissions may be prevented with good outpatient care (Oddone 1996) and various studies have shown that disease management programs can reduce HF admissions by up to 80% (Rich 1995)(Cline 1998)(Stewart 1999)(Blue 2001)(McDonald 2002). HF clinics and disease management programs can provide early identification and intervention of symptom progression; patient education to increase adherence; identify contributors to HF progression; manage medical, socioeconomic, and psychologic factors that contribute to exacerbations; and establish ways to document and monitor quality of care (Hauptman 2008). Thus, the Heart Failure Society of America (HFSA) and the American College of Cardiology/American Heart Association (ACC/AHA) recognize the potential usefulness and advocate the use of disease management systems as a Class I recommendation for patients at high risk for admission or clinical deterioration (Hunt AHA 2009)(Hauptman 2008).

Despite early outpatient interventions, which often include increasing oral diuretic therapy, patients often do not respond and require re-hospitalization. Several studies have shown that outpatient intravenous (IV) diuretic therapy is safe and cost effective, may stabilize evolving deterioration and prevent hospital admission (Ryder 2007)(Durango 2006)(Hebert 2011). Ryder et al, found that 72% of patients stabilized following IV diuretics, did not require admission and were not complicated by hypotension or electrolyte abnormalities (Ryder 2007). A prospective study in Ireland found that the outpatient IV furosemide program potentially prevented 115 admissions or ER visits over a 3-month period, with estimated cost saving of nearly 3 million dollars (Hebert 2011).

Although there have been a number of small positive studies indicating the effectiveness of outpatient IV diuretics, to date, there has been no randomized controlled trial. Furthermore, clinical improvement may have been from the multidisciplinary support, guidance, close follow-up and benefits provided by the HF clinic rather than from the IV furosemide administration.

We believe that many acute decompensations of HF could be managed in the outpatient setting by a specifically trained health care team, and may potentially reduce costs by reducing ED utilizations and/or hospitalizations. Our goal is to further evaluate the feasibility, efficacy and safety of outpatient IV diuretic therapy in treatment of acute decompensated heart failure (ADHF) in a randomized controlled, double blind fashion.

**II. SPECIFIC AIMS**

1. **Primary endpoints** include 30-day hospital readmission for heart failure and death.
2. **Secondary endpoint** is to evaluate the feasibility, safety and outcomes of outpatient IV diuretic therapy in treating ADHF.
   - Clinical stability defined as resolution of acute symptoms and physical signs, with a return to baseline status, including dry baseline weight. Q QQuality of Life will be quantified using Kansas City Cardiomyopathy Questionnaire (KCCQ)
   - All episodes of clinical deterioration and adverse effects including hypotension (SBP <90 mmHg), creatinine elevation (>25% baseline or >2x baseline), BNP, hyponatremia (Na < 130), hypokalemia (K <3.5).

**III. SUBJECT SELECTION**

New York Methodist Hospital is a community-based teaching hospital, serving one of the most diverse counties in the United States. There are approximately 60-70 patients admitted monthly for HF, which accounts for 2.5% of all hospital admissions. The average length of stay is 4.5 days though Medicare guidelines recommend 3.4 days for HF patients. Furthermore, the 30-day readmission rate for patients with a HF is 26%, which is above the national average of 24.5%.

Patients presenting to the emergency room for symptoms of HF [Table 1] including shortness of breath, edema, orthopnea, weight gain, etc. or chest x-ray demonstrating pleural effusion, pulmonary congestion, or cardiomegaly will be evaluated for enrollment. Consent will be obtained prior to randomization.

1. **Inclusion Criteria**

- Patients from emergency department being evaluated for heart failure over 18 years old and patients being discharged for ADHF
- Known history of systolic and diastolic dysfunction of greater than 6 weeks
- NYHA Class II-IV
- Heart failure as defined in [Table 1]. One symptom must be present at time of screening and one sign must be present in the last 12 months
- Elevated pro-BNP ≥ 360 pg/ml and not explained by any other etiology
- Consentable and willing to comply to scheduled visits and phone calls

| **Table 1. Criteria for Diagnosing Heart Failure** | |
| --- | --- |
| SYMPTOMS (at least 1 must be present at time of screening): | SIGNS (at least 1 in last 12 months): |
| - Paroxysmal nocturnal dyspnea - Orthopnea - Dypsnea on mild or moderate exertion | - Any rales post cough - Jugular venous pressure ≥ 10 cm H20 - Lower extremity edema - CXR demonstrating pleural effusion, pulmonary congestion, or cardiomegaly |

1. **Exclusion Criteria**

- Systolic blood pressure <90 mmHg
- Signs of significant respiratory distress, according to the discretion of the investigator.
- Implantable cardioverter-defibrillator within 15 days, cardiogenic shock or volume depletion
- Chronic dialysis
- Acute renal failure defined as creatinine > 2 x baseline
- Severe systemic illness with life expectancy judged less than three years
- Chronic pulmonary disease requiring home O2, oral steroid therapy or hospitalization for exacerbation within 12 months, or significant chronic pulmonary disease in the opinion of the investigator
- Primary hemodynamically significant uncorrected valvular heart disease, obstructive, or regurgitant, or any valvular disease expected to lead to surgery during the trial.
- Known infiltrative or hypertrophic cardiomyopathy or know pericardial constriction
- Atrial fibrillation with resting heart rate >90 bpm
- Myocardial infarction in past 90 days
- Percutaneous coronary intervention in past 30 days
- Heart transplant recipient or currently implanted left ventricular assist device
- Stroke in past 90 days
- No acute infection especially requiring IV antibiotics
- Allergy to lasix
- Known chronic hepatic disease, defined as aspartate aminotranferase (AST) and alanine aminotranferase (ALT) levels > 3.0 times the upper limit of normal
- Non-verbal patients, patients who cannot speak or understand English, patients with dementia and psychiatric illness, patients who are blind or deaf and patients who are transferred to different hospital will be excluded.

**IV. SUBJECT ENROLLMENT**

- 1. **Recruitment Protocol**

The principal investigator and research coordinator/designee in coordination with the ER physicians and cardiologists will have the responsibility for case finding and subject recruitment. The coordinator will conduct chart review, while complying with local HIPAA requirements, to identify potentially eligible subjects. Patients over 18 years of age, being evaluated for heart failure exacerbation will be enrolled from emergency department and patients being discharged from the hospital for ADHF. Coordinator will obtain consent prior to enrollment.

- 1. **Stratification**

Eligible patients from the ER will be randomized 1:1:1 [Figure 1] into standard of care (group 1), double blind treatment group using IV bolus furosemide (up to 150 mg) followed by continuous infusion (40-80 mg/hr) according to the physician’s judgement (group 2) or IV bolus furosemide followed by placebo (group 3). Drips will be concentrated [1-5 mg/ml] by pharmacy to minimize fluid intake. Pt randomized into group 2 and 3 will follow up at the HF clinic after initial therapy in the ER. Infusions may be repeated up to 3 times weekly as necessary. The randomization sequence will be generated by a computer-generated randomization plan.

Patients enrolled from discharge or from outpatient clinic will be randomized into group 2 or group 3. IV infusion of furosemide/placebo will be given on the discretion of the physician.

- 1. **Blinding**

Subjects, nurses and treating physicians will be blinded to whether the subjects are receiving continuous IV furosemide (group 2) or continuous IV placebo (group 3). All patients will receive IV furosemide bolus according to the physician’s discretion. The pharmacist and coordinator will not be blinded to help administer the appropriate medication. Because the trial will be double-blinded, safety laboratory tests will be performed for each subject for the duration of the trial, regardless of the treatment arm, and will be monitored by the research coordinator. Similarly, monitoring of potential side effects will be continuous and irrespective of treatment assignment.

- 1. **Baseline Visit and Randomization**

After consent is obtained and the patient is randomized, the subject will be either treated as standard of care (group 1) or receive continuous IV furosemide [40-80 mg/hr] (group2) vs placebo (group3). Bolus IV furosemide [Up to 150 mg] will be given in the emergency department prior to randomization . Patients will have baseline laboratory evaluation including metabolic panel and BNP.

Patients enrolled from discharge will follow up at the HF clinic and IV bolus infusion of furosemide/placebo will be given on the discretion of the physician. Baseline echo and a repeat echo at 3 months will be performed to assess LVEF and diastolic parameters. Patients will be monitored for changes such as dizziness, weakness, hypotension, ototoxicity, or palpitations after the IV push. The duration of infusion will be for 3-4 hours and may be repeated for up to 3 times a week as necessary. A minimum of 2 visits per week will be performed. Duration of visits will last for 30 days. Repeat metabolic panel will be done at the end of infusion.

**V. STUDY PROCEDURES**

1. **Description of the Study Medication**

Continuous IV furosemide will be prepared by the pharmacist involved in the study in a concentrated dose so that minimal IVF will be given especially to the placebo arm. Both the IV furosemide drip and placebo drip will be in accordance with federal regulations.

1. **Randomization Procedures**

Subjects will be assigned in the order they are enrolled into the study, to receive the allocated treatment according to a computer-generated randomization plan. Once a subject has been assigned a Treatment Allocation Code, the subject will remain on the same treatment allocation code for the duration of the study. The standard of care arm will be admitted or discharged from the emergency room based on the discretion of the physicians involved. If admitted, the subject will be treated in the usual manner.

1. **Drug Administration**

Appropriate furosemide or placebo drip will be dispensed at randomization and as often as the research physician in coordination with the primary physician decides based on the patient’s clinical progression, up to 3 times a week.

1. **Non Medical Interventions**

All patients randomized to lasix infusion or placebo will be educated on the signs and symptoms of HF, medications, diet, and activity for HF. In addition, each patient will receive oral and written instructions on sliding-scale oral diuretics, primarily furosemide, for weight gain of > 2 pounds as well as instructions for recording daily weights. For weight gain of > 5 pounds above baseline weight, or worsening symptoms refractory to oral diuretics, patients will receive oral and written instructions regarding open access to the infusion center during clinic hours. The standard of care arm will receive the usual education provided by the hospital staff.

1. **Concomitant Medications**

Subjects will be treated with other medications at the discretion of their cardiologist and/or primary care provider. At study visits, current medications will be recorded on the study forms.

1. **Indications for Withdrawal From the Study**

Subject may refuse to continue in the study at any time.

All protocol-specified visits and follow-up procedures should be performed for every subject enrolled in the trial. If the subject refuses to continue with the study visits, every attempt will be made to continue contact by telephone, written communication, or record review to determine if outcome events have occurred, unless the subject specifically refuses such follow-up. The reason for withdrawal will be documented for all subjects withdrawn from the study.

1. **Study Completion**

A subject will be considered to have completed the study if he/she has completed follow-up until the end of the trial period 3 months, undergoes transplantation, or dies. All subjects will be followed for 3 months. The aim of this study is to enroll 165 patients as a pilot study with 3 months follow up for each patient.

1. **Measurements (including questionnaire QoL, etc)**

- Treatment efficacy will be assessed by NYHA class; change in body weight and BNP levels; need for hospitalization.
- Safety outcome measures will include electrolyte abnormalities, renal function, hospitalization for any reason and mortality.
- The primary goals of heart failure management are improving patient function, slowing disease progression, and improving quality of life. Quality of Life will be quantified using Kansas City Cardiomyopathy Questionnaire (KCCQ) and the Patient Health Questionnaire (Depression Scale). Each assessment will be done on initial visit and in 30 days, typically 12-15 mins each.
- Clinical characteristics such as age, sex, race, education level, insurance status, ejection fraction, NYHA level, hypertension, diabetes, medications, and laboratory values will be analyzed to identify clinical variables.
- Echo will be performed on presentation and repeated in 3 months to assess LV function and diastolic parameters.

**X. REFERENCES**

1. McMurray JJ, Stewart S. Epidemiology, aetiology and prognosis of heart failure. Heart 2000;83:596–602.
2. Jencks SF, Williams MV, Coleman EA. N Engl J Med 2009;360:1418-28.
3. Roger VL, Go AS, Lloyd-Jones DM, et al. *Circulation*. 2011;123:e18-e209
4. Ashton CM, Kuykendall DH, Johnson ML, et al. *Ann Intern Med.* 1995;122:415-421.
5. Vinson JM, Rich MW, Sperry JC, Shah AS, McNamara T. Early readmission of elderly patients with congestive heart failure. *J Am Geriatr* *Soc*. 1990;38:1290 –1295.
6. Van Jaarsveld C, Ranchor AV, Kempen G, et al. The European Journal of Heart Failure 8 (2006) 23 – 30
7. Andrews, R.M. *The National Hospital Bill: The Most Expensive Conditions by Payer, 2006. HCUP Statistical Brief #59*. September 2008. Agency for Healthcare Research and Quality, Rockville, MD.
8. Oddone EZ, Weinberger M, Horner M, et al., for the Veterans Affairs Cooperative Studies in Health Services Group on Primary Care and Hospital Readmissions. Classifying general medicine readmissions. Are they preventable? J Gen Intern Med. 1996;11:597–607.
9. Rich MW, Beckham V, Wittenberg C, Leven CL, et al. A multidisciplinary intervention to prevent the readmission of elderly patients with congestive heart failure. N Engl J Med 1995;333:1190–5.
10. Cline CMJ, Israelsson BYA, Willenheimer RB, et al. Cost effective management programme for heart failure reduces hospitalisation. Heart 1998;80:442–6.
11. Stewart S, Marley JE, Horowitz JD. Effects of a multidisciplinary, homebased intervention on unplanned readmissions and survival among patients with chronic congestive heart failure: a randomised controlled study. Lancet 1999;354:1077–83.
12. Blue L, Lang E, McMurray J, et al. Randomised controlled trial of specialist nurse intervention in heart failure. Br Med J 2001;323:715–8
13. McDonald K, Ledwidge M, Cahill J, et al. Heart failure management: multidisciplinary care has intrinsic benefit above the optimization of medical care. J Card Fail 2002;8:3:142–8.
14. Hauptman PL, Rich MW, Heidenreich PA, et al. J Cardiac Fail 2008;14:801e815
15. Hunt SA, Abraham WT, Chin MH, et al. 2009 focused update incorporated into the ACC/AHA 2005 Guidelines for the Diagnosis and Management of Heart Failure in Adults: a report of the American College of Cardiology Foundation/American Heart Association Task Force on Practice Guidelines: developed in collaboration with the International Society for Heart and Lung Transplantation. *Circulation.* 2009;119:e391– e479.
16. Ryder M, Murphy NF, McCaffrey D, et al. European Journal of Heart Failure 10 (2008) 267–272
17. Durango LF, Katz SD, Li H-K, Wencker D. Abstract 1891: continuous furosemide infusion is safe and effective in outpatient treatment of refractory heart failure. Circulation. 2006;114:II_375–II_376.
18. Hebert K, Dias A, Franco F, et al. Congest Heart Fail. 2011 Nov-Dec;17(6):309-1


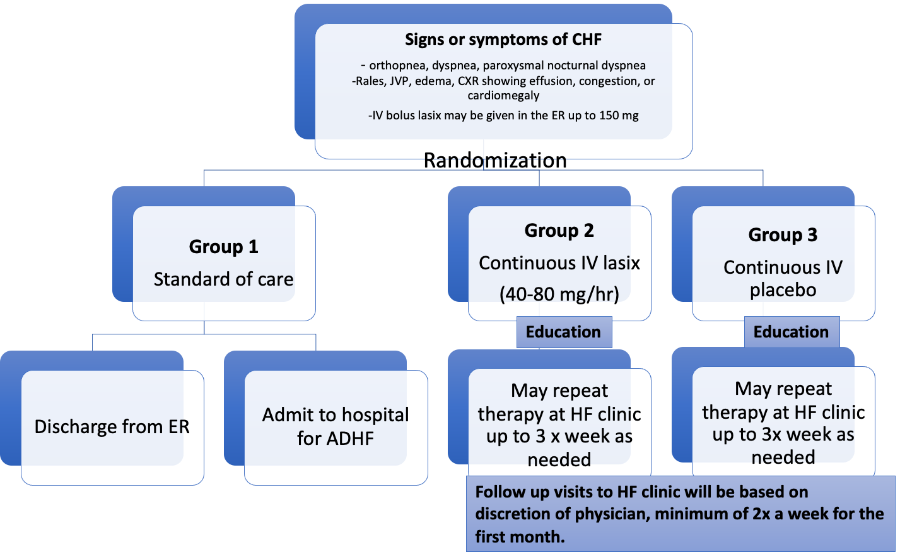


**Figure 1**. Flow diagram of procedure for outpatient diuretic therapy.
